# Novel Indoor Residual Spray Insecticide With Extended Mortality Effect: A Case of SumiShield 50WG Against Wild Resistant Populations of *Anopheles arabiensis* in Northern Tanzania

**DOI:** 10.9745/GHSP-D-18-00213

**Published:** 2018-12-27

**Authors:** Eliningaya Kweka, Aneth Mahande, Johnson Ouma, Wycliffe Karanja, Shandala Msangi, Violet Temba, Lucille Lyaruu, Yousif Himeidan

**Affiliations:** a Department of Medical Parasitology and Entomology, School of Medicine, Catholic University of Health and Allied Sciences, Mwanza, Tanzania.; b Mosquito Section, Division of Livestock and Human Health Disease Vector Control, Tropical Pesticides Research Institute, Arusha, Tanzania.; c Mabogini field station, Division of Livestock and Human Health Disease Vector Control, Tropical Pesticides Research Institute, Moshi, Tanzania.; d Africa Technical Research Centre, Vector Health International Ltd., Arusha, Tanzania.

## Abstract

**Background:**

Resistance of malaria vectors to different classes of insecticides has been reported in malaria-endemic areas. Identifying new indoor residual spray (IRS) compounds that are effective against resistant vector populations is a high priority in managing insecticide resistance.

**Method:**

A biological efficacy trial was conducted in the field from August 2016 to February 2017 to determine the efficacy of SumiShield 50WG, a new insecticide class, against wild *Anopheles arabiensis*. Indoor surfaces of 20 houses in Mabogini ward in the rural district of Moshi in northern Tanzania were sprayed with SumiShield 50WG. Bio-efficacy monitoring was conducted monthly for 6 months after the spray application. In addition, susceptibility tests were conducted by exposing mosquitoes to papers treated with permethrin 0.75%, pirimiphos-methyl 0.25%, and clothianidin 2% (SumiShield 50WG). Representatives from each household included in the study were surveyed about possible side effects or problems faced since the spray. Regression probit analysis was used to calculate knock-down times while the chi-square test was used to compare the mortality effect for mosquitoes.

**Results:**

The SumiShield 50WG insecticide maintained optimal efficacy in the field setting for the duration of the 6-month study period, with 100% mortality of mosquitoes by 144 to 168 hours post-exposure to treated surfaces. Susceptibility tests showed some variation in tolerance to the tested insecticide-treated papers, particularly between SumiShield 50WG and pirimiphos-methyl. The knock-down times for 50% and 95% of the mosquitoes when exposed to SumiShield 50WG-treated test paper were 45.81 minutes and 83.85 minutes, respectively, and 67.77 minutes and 105.81 minutes, respectively, for the pirimiphos-methyl-treated papers. There were no short-term adverse side effects reported by households sprayed with SumiShield 50WG.

**Conclusion:**

The findings of this study suggest that SumiShield 50WG is a viable IRS insecticide for malaria vector control in Tanzania, especially in areas where pyrethroid resistance is a concern.

## Abstract (precis)

The new SumiShield 50WG insecticide, which possibly has longer duration of effectiveness than other indoor residual spray (IRS) formulations, has potential as an alternative IRS product for malaria vector control, particularly where resistance to other formulations has developed.

## INTRODUCTION

Despite the general decline in malaria transmission, malaria remains a major cause of morbidity and mortality in many tropical countries including Tanzania.^1^^,^^2^ Malaria vector control campaigns have been advocating the use of indoor residual spray (IRS) and long-lasting insecticidal bed nets (LLINs) to reduce malaria transmission.^3^^–^^5^ The scale-up of LLINs in Tanzania has been successful, with coverage of 75% and up to 100% in some parts of the country, mostly in the Lake Zone regions (Kagera, Mara, Mwanza, and Shinyanga).^6^^–^^8^ However, the IRS program in Tanzania has faced many challenges, as is the case in other countries around the world.

IRS programs have been implemented worldwide since the 1950s and have been shown to decrease vector density, leading to a decline in malaria transmission in various settings.^9^^,^^10^ The different wall types in malaria-endemic areas, however, have hindered the performance and effectiveness of sprayed insecticides.^11^^,^^12^ Another challenge is that malaria vectors have developed, and continue to develop, resistance against most classes of insecticides sprayed. Studies conducted in several malaria-endemic countries including Tanzania show that resistance has developed against organophosphates,^13^^,^^14^ pyrethroids,^13^^,^^15^^,^^16^ and carbamates.^13^^,^^17^ To sustain the gains achieved in malaria control and ensure continued success of IRS, programs must identify compounds from new classes of insecticides with long-lasting efficacy and ensure they are used judiciously and according to the Global Plan for Insecticide Resistance Management (GPIRM) that was coordinated by the World Health Organization's (WHO's) Global Malaria Programme.^18^

To sustain the gains achieved in malaria control, programs must identify compounds from new classes of insecticides and ensure they are used judiciously.

In searching for an innovative insecticide replacement, a new IRS compound called SumiShield 50WG, containing the neonicotinoid insecticide clothianidin, has been developed. This formulation, manufactured by Sumitomo Chemical Co. Ltd, Tokyo, Japan, is expected to retain its bio-efficacy for much longer than many other existing IRS products. This study evaluated the bio-efficacy of SumiShield 50WG against wild resistant populations of *Anopheles arabiensis* in northern Tanzania.

This study evaluated the bio-efficacy of SumiShield 50WG, a new IRS compound, against wild resistant populations of *Anopheles arabiensis* in northern Tanzania.

## METHODS

### Study Setting

This study was conducted in Mabogini ward in the rural district of Moshi, which is located on the southern foothills of Mount Kilimanjaro in northern Tanzania.^19^ In this area, rice is grown on more than 400 hectares of land under an irrigation scheme, making the area conducive for breeding malaria vectors. Malaria transmission occurs throughout the year albeit with low parasitemia^20^^–^^23^ and a low entomological inoculation rate.^24^ The predominant mosquito species in the area are *An. arabiensis* and *An. funestus*.^4^^,^^16^^,^^25^^–^^27^ For a detailed description and map of the area, refer to Lowassa and colleagues (2012).^26^

Twenty houses with different types of wall surfaces (i.e., walls made of brick, burnt brick, or mud) sprayed with SumiShield 50WG were selected for 6 months of follow-up. The spray application was conducted over a 4-day period, and the houses were at least 10 meters apart. This study took place from August 2016 to February 2017.

### Calibration of the Sprayers

The spray team used in total 6 8-liter Hudson X-Pert sprayers. The nozzles of the sprayers were checked first per guidelines from the WHO Pesticide Evaluation Scheme (WHOPES).^28^ The flow rate of the constant flow valve used for the 6 sprayers ranged between 760 to 790 ml/minute at a tank pressure of 55 psi. This range was within the WHOPES-recommended flow rate of 681 to 832 ml/minute. The hardness of the water used in the field ranged from 0.0 to >4.0 mg/m^3^, and the pH from 5.5 to 7.0 mg/m^3^, as measured at different points over the 4-day spray period. Any water used for mixing of the products or for washing the sprayer was filtered using a double layer of new polyester cloth and a water sieve.

### Indoor Residual Spraying Procedures

Indoor residual spraying was conducted following WHO standard procedures described elsewhere.^29^ Briefly, SumiShield 50WG was applied according to label claims with a targeted dosage of 300 mg active ingredient per m^2^. Household items such as furniture and other utensils were gathered into the middle of rooms to expose the wall surfaces for spraying. All room walls in each house were uniformly sprayed. The time spent to spray each house depended on the number and size of the rooms as well as the house content, but in general the spray operators did not take more than 30 minutes per house.

### Assessment of Spray Quality and Uniformity

Two methods were used to assess the spray accuracy: (1) calculating the actual volume sprayed by weighing the sprayer before and after spraying and determining the area sprayed, and (2) analyzing the active ingredient content sprayed on filter paper attached to the surface sprayed as described by WHO.^29^

For the first method, the empty sprayers were weighed and the weight recorded. Water and the insecticide formulation were then added and weighed before and after spraying each house. The volume sprayed was determined by subtracting the weight of sprayer and contents after spraying from the weight of the depressurized sprayer and contents before spraying. The surface sprayed was measured for the selected houses. The sprayed dose in mg per m^2^ was calculated as *[sprayed volume x mg active ingredient per liter]/surface area sprayed*.

For the second method, 3 points were selected in 1 room in each sprayed house and labeled with masking tape as low (2 feet from the floor), middle (4 feet from the floor), and high (6 feet from the floor). Three filter papers were then fixed at these 3 points on a separator at a distance from the wall to prevent insecticide running down the wall and contaminating the paper. After spraying the surface, chemical analysis of the filter papers was carried out in 1 of 2 laboratories: Health & Crop Sciences Research Laboratory (HCRL), Takarazuka, Japan, or the Africa Technical Research Centre (ATRC), Arusha, Tanzania.

### Cone Bioassays

Standard WHO cone bioassays were conducted on the walls of treated houses at monthly intervals for 6 months after the spray application to assess residual efficacy. The F1 *An. arabiensis* offspring reared from field-collected larvae were used in cone bioassays. Ten 4-day-old unfed female mosquitoes per cone were exposed to the walls of houses sprayed with SumiShield 50WG. At monthly intervals, in each selected room a total of 40 mosquitoes were exposed for 30 minutes and collected in paper cups as 4 replicates of 10 mosquitoes per cup.^29^ In all assays in the field, knocked-down and live mosquitoes were recorded at 60 minutes and mortality was observed at intervals of 24 hours post-exposure, up to 168 hours (7 days) post-exposure. After exposure, the female mosquitoes were placed in 150 ml cups (10 mosquitoes per cup), with sugar solution provided, and maintained in a climatic chamber for 24 hours at 27° Celsius ± 2° Celsius and 80% ± 10% relative humidity. Field experiments were conducted monthly to monitor the bio-efficacy of insecticides in each treated house included in the study.

Standard WHO cone bioassays were conducted on the walls of treated houses at monthly intervals for 6 months after the spray application to assess residual efficacy.

### Susceptibility Tests

Susceptibility tests were conducted using the standard WHO protocol.^30^ The treated papers used were permethrin 0.75% (treated with technical-grade permethrin with cis:trans ratio of 40:60, Lot: GBPRTG052E), pirimiphos-methyl 0.25% (treated with technical-grade pirimiphos-methyl, Lot: SZBC010XV), and clothianidin 2% (treated with SumiShield 50WG, Lot: 16940015056Y). Mosquito larvae were collected from lower Moshi rice-irrigated fields and reared in the insectary until they emerged and reached 4 days old. They were exposed to insecticide-treated papers for 1 hour, and mortality was recorded at 24 hours post-exposure and, for the clothianidin-treated papers only, up to 168 hours post-exposure. A total of 600 mosquitoes were tested for each insecticide.

### Chemical Analysis of Filter Papers

The filter papers for residual dose monitoring were extracted in organic solvents and analyzed for clothianidin content by high performance liquid chromatography. The active ingredient contents were divided by the area of the sprayed filter paper to obtain the dose per m^2^. The chemical content in the filter papers was analyzed by either HCRL in Japan or ATRC in Tanzania. The laboratories used the same protocol that had been validated using filter paper samples treated with the target dose at HCRL.

### Side Effects

The study team surveyed head of households from the 20 treated houses to ask about all possible side effects or problems faced since the day the house was sprayed. All mentioned cases, if any, were recorded.

### Data Analysis

Data were analyzed using PASW (Predictive Analytics Software) Statistics version 18 (SPSS Inc., Chicago, IL). Descriptive tests were deployed for data analysis to obtain the confidence intervals and mean of difference. Microsoft Excel 2016 spreadsheets were used to calculate percentage mortalities for field mosquitoes. Regression probit analysis was used to calculate the KDT_50_ (knock-down time for 50% of the mosquitoes) and KDT_95_ (knock-down time for 95% of the mosquitoes) while the chi-square test was used to compare the mortality effect for mosquitoes 24 hours post-exposure to the 3 insecticides in the susceptibility tests.

## RESULTS

### Spray Quality and Uniformity

**FIGURE 1:**
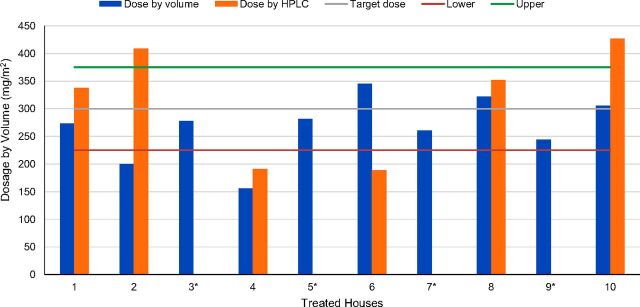
Assessment of Spray Accuracy by Volume Measurement and Chemical Analysis, Moshi, Tanzania Abbreviation: HPLC, high performance liquid chromatography. Due to constraints with performing analysis of the collected specimens, assessment of spray quality was performed on only 10 of the 20 treated houses. * HPLC data are missing for 4 of the treated houses.

Analysis of the chemical content of the filter papers for the sprayed houses showed that the correct dose was sprayed, with the average being 363.4 mg/m^2^ (Table 1). The acceptable range is 300 mg/m^2^ ± 25%. The residual doses obtained by chemical analysis and by volume measurement were generally similar from one house to another (Figure 1).

**TABLE 1.** Active Ingredient of Clothianidin (SumiShield 50WG) Sprayed on Filter Papers^a^

Formulation	Clothianidin Content (mg/m^2^)					% RSD
	Upper	Middle	Lower	Mean	SD	
SumiShield 50WG	334.5	384.9	368.7	363.4	165.4	45.5

Abbreviations: RSD, relative standard deviation; SD, standard deviation.

a Results were pooled from tests of 12 houses performed by the Health & Crop Sciences Research Laboratory in Takarazuka, Japan, and 16 houses performed by the African Technical Research Centre in Arusha, Tanzania.

### Susceptibility Tests

**FIGURE 2:**
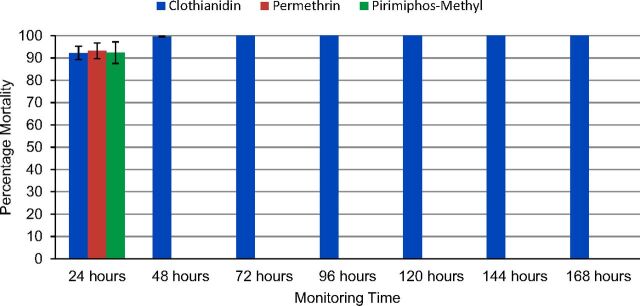
Susceptibility of *Anopheles arabiensis* to Clothianidin (SumiShield 50WG), Permethrin, and Pirimiphos-Methyl by Post-Exposure Time^a^ ^a^ Only clothianidin-treated papers were monitored beyond 24 hours because it has extended mortality effects.

Susceptibility tests for the wild population of *An. arabiensis* showed some variation in tolerance to the tested insecticide-treated papers, particularly between SumiShield 50WG and pirimiphos-methyl. Specifically, the knock-down times for 50% and 95% of the mosquitoes when exposed to the SumiShield 50 WG-treated test paper were 45.81 minutes and 83.85 minutes, respectively (Table 2). The permethrin-treated papers showed similar results. However, the pirimiphos-methyl-treated papers had higher knock-down times: 67.77 minutes for 50% of the mosquitoes and 105.81 minutes for 95% of the mosquitoes. The 24-hour mortality was not statistically significant among the 3 insecticides tested (Χ*^2^*=0.0942; *P*=.95) (Figure 2). The susceptibility of wild population of *An. arabiensis* to permethrin and pirimiphos-methyl was monitored for 24 hours only, but for clothianidin mortality was monitored for 168 hours as it has additional delayed mortality effect beyond the first 24 hours exposure. At each 24-hour period from 48 hours post-exposure to 168 hours post-exposure, 100% of the mosquitoes exposed to SumiShield 50WG were fully susceptible to the insecticide (Figure 2).

**TABLE 2.** Knock-Down Times (in minutes) for the Insecticide-Exposed Mosquitoes, by Insecticide Type

Insecticide	KDT_50_ (95% CI)	KDT_95_ (95% CI)
SumiShield 50WG	45.81 (44.34, 47.35)	83.85 (80.81, 87.24)
Permethrin	47.73 (45.79, 49.75)	85.77 (82.45, 89.45)
Pirimiphos-methyl	67.77 (64.84, 70.88)	105.81 (101.46, 110.61)

Abbreviations: CI, confidence interval; KDT, knock-down time.

Note: Mosquitoes were exposed to each insecticide for 1 hour.

100% of mosquitoes exposed to SumiShield were susceptible within 168 hours of exposure.

### Residual Efficacy of SumiShield 50WG From Bioassays

**FIGURE 3:**
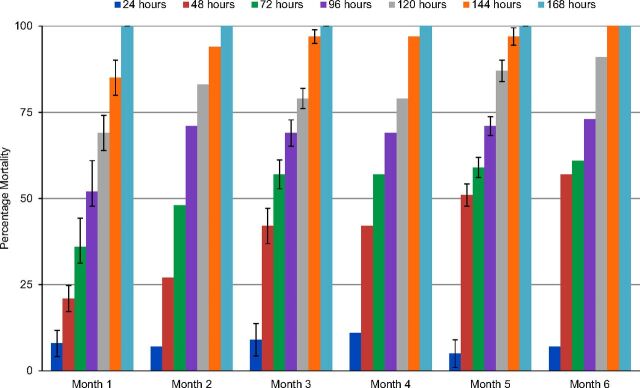
Residual Efficacy of SumiShield 50WG: Percentage Mortality of Mosquitoes After Exposure for 30 Minutes in Cone Bioassays on Treated Surfaces

The residual efficacy of SumiShield 50WG was observed to be constant for 6 months, with 100% mortality of mosquitoes when tested 168 hours after exposure to treated surfaces including mud and concrete (Figure 3).

### Side Effects and Community Acceptability of SumiShield 50WG

In all houses sprayed, none of the household members was recorded to have had any adverse effects (e.g., sneezing or itching) related to SumiShield 50WG spray application up to 6 months later. All community members whose houses were involved were interviewed (n=20), and all found the SumiShield 50WG trial application acceptable and reported being willing to participate in the next round of spraying.

## DISCUSSION

In this study, the new SumiShield 50WG IRS compound was found to be effective against field populations of *An. arabiensis*. While initial mortality of exposed mosquitoes was low, we observed and documented an increasing kill effect over time, reaching 100% mortality at 144 to 168 hours post-exposure. This new compound holds great promise in vector control as it has higher efficacy than other IRS formulations.^31^^–^^34^ In addition, the residual efficacy of 6 months shown for SumiShield 50WG is higher than reported for other compounds in previous studies in malaria-endemic regions.^35^^–^^37^ The bio-efficacy observed against the wild-resistant population of *An. arabiensis* is similar to other studies using SumiShield 50WG conducted in Africa and Asia at the community level.^32^^–^^34^

SumiShield 50WG holds great promise in vector control as it has higher efficacy than other IRS formulations, including long-lasting residual efficacy.

The efficacy of SumiShield 50WG was shown to be above the WHO-recommended mortality cut-off point of 80% for the entire 6-month trial period, and no decline in mortality was observed throughout this period against the wild-resistant population of *An. arabiensis*. These findings demonstrate a better efficacy of SumiShield 50WG than that previously observed in studies with pyrethroids, organophosphates, carbamates, and organophosphates.^5^^,^^13^^,^^16^^,^^38^^–^^40^ The efficacy of SumiShield 50WG against wild populations of *An. arabiensis* is attributed to it being non-repellent—unlike other insecticides such as pyrethroids—which increases the possibility of the vector getting the peak lethal dose of the insecticide from the treated surfaces. Similar responses have been shown by other studies evaluating SumiShield 50WG.^32^^–^^34^

The bio-efficacy and residual effect of SumiShield 50WG reported from this field study in Tanzania has shown that the product retains maximum mortality efficacy for 6 months while other IRS compounds used in Tanzania were found to have sharply decreased mortality from the fourth to the sixth month.^5^^,^^29^^,^^32^^–^^34^^,^^40^ Therefore, SumiShield 50WG provides the potential for longer residual protection as it consistently maintains its residual activity once applied. With complete coverage, SumiShield 50WG provides a lethal dose to mosquitoes that land on sprayed surfaces, with complete mortality effects observed at 144 to 168 hours after exposure.^2^^,^^29^^,^^32^^–^^34^ SumiShield 50WG was very effective on both concrete and mud surfaces tested under the field conditions of the lower Moshi region of Tanzania.

## CONCLUSION

The findings of this study suggest that SumiShield 50WG is a suitable alternative IRS insecticide for rotational use in malaria vector control in Tanzania, including all endemic areas, particularly where resistance to the existing organophosphorus IRS formulation has started to develop.

## Notes

**Peer Reviewed**

**Funding:** Sumitomo Chemical Co., Ltd.
